# Prospective Exploratory Phase I Clinical Trial Assessing the Safety of Preoperative Marking for Small Liver Tumors

**DOI:** 10.7759/cureus.50603

**Published:** 2023-12-15

**Authors:** Daisuke Takei, Shintaro Kuroda, Tsuyoshi Kobayashi, Hiroaki Mashima, Hiroyuki Tahara, Masahiro Ohira, Hiroshi Aikata, Keigo Chosa, Yasutaka Baba, Hideki Ohdan

**Affiliations:** 1 Department of Gastroenterological and Transplant Surgery, Graduate School of Biomedical and Health Sciences, Hiroshima University, Hiroshima, JPN; 2 Department of Gastroenterology and Metabolism, Hiroshima University, Hiroshima, JPN; 3 Department of Diagnostic Radiology, Hiroshima University, Hiroshima, JPN; 4 Department of Diagnostic Radiology, Saitama Medical University International Medical Center, Hidaka, JPN

**Keywords:** resection, safety, surgery, preoperative marking, liver tumor

## Abstract

Background

Small tumors in liver cirrhosis are difficult to distinguish using intraoperative ultrasonography. In addition, preoperative chemotherapy for metastatic liver cancer may diminish tumor size, thus making tumors difficult to identify intraoperatively. To address such difficulties, we devised a method to mark liver tumors preoperatively to facilitate intraoperative identification. This study aimed to investigate the safety of a preoperative liver tumor marking method.

Methodology

This exploratory prospective clinical trial included patients with liver tumors measuring ≤20 mm requiring resection. Preoperative marking was performed by placing a coil for embolization of blood vessels near the tumor using either the transcatheter or percutaneous approach. The tumor was identified and resected by intraoperative ultrasonography based on the marker. The study was registered in the University Hospital Medical Information Network Clinical Trials Registry (UMIN000028608).

Results

Overall, 19 patients (9 with primary liver cancer and 10 with metastatic tumors) were recruited. The transcatheter and percutaneous methods were used in 13 and 6 patients, respectively. Marking was not possible in two patients in the transcatheter group because the catheter could not be guided to the vicinity of the tumor. There were no marking-related complications. Hepatectomy was performed in all but one patient who was not fit for hepatectomy owing to the development of a metastatic liver tumor. The markers were adequately identified during hepatectomy. Additionally, there were no difficulties in the surgical procedure or postoperative complications.

Conclusions

Preoperative marking with embolization coils can be performed safely for intraoperative identification of liver nodules.

## Introduction

Liver surgery is a primary treatment modality for hepatocellular carcinoma (HCC) and colorectal liver metastasis, markedly reducing mortality and morbidity over the last decade [1-4]. Advances in diagnostic imaging such as computed tomography (CT) and magnetic resonance imaging (MRI) have led to earlier identification of liver malignancies such as HCC, with an increase in the incidence of small tumors. These tumors had previously not been considered surgical targets but are now regarded as an indication for hepatectomy [5]. In the fourth Japan Society of Hepatology-HCC guidelines, surgical resection is recommended as the first-line therapy for solitary HCC, regardless of size, with Child-Pugh A/B liver function and without extrahepatic metastasis or vascular invasion [6]. In addition, an increasing number of cases in which liver tumors that have shrunk due to successful chemotherapy, such as liver metastasis from colorectal cancer, are being resected [7].

Gastrointestinal tumors may recur after completion of chemotherapy. Particularly, complete response (CR) on imaging and pathological diagnosis could be inaccurate among patients with primary or metastatic liver cancer. Therefore, several patients who achieve CR on imaging still require exploratory surgery. Moreover, despite improvements in the treatment and prognosis of HCC, recurrence remains a clinical challenge [8]. The number of patients with micro-recurrent HCC who will undergo resection is predicted to increase further [9].

Liver tumors are usually identified using intraoperative ultrasound (IOUS), and a hepatectomy is accordingly performed to include the liver tumor. However, in our experience, small liver tumors are often unidentifiable on IOUS. In such cases, excision is usually performed based on the positional relationship between the vessel running in the liver and the liver tumor determined from preoperative imaging tests. Hence, there is always a possibility of a wider extent of hepatectomy, and, in rare cases, the tumor may not even be resected. This is a major disadvantage for patients and a major challenge in current hepatectomy techniques.

To date, only one clinical study on percutaneous CT-guided marking for metastatic liver tumors has been reported [10], and, to our knowledge, no reports of transcatheter marking in liver resection have been published. Preoperative marking is a standard practice for deep-seated tumors in other solid organs, such as the lung [11]. Furthermore, the usefulness of coil marking for lung tumors has been reported [12,13]. Preoperative marking via endoscopic instillation or clipping for gastrointestinal tumors is also common. Originally, we would have liked to conduct a Phase II study to confirm the usefulness of the procedure; however, as mentioned above, only a few case series for percutaneous marking have been reported [10], and, to our knowledge, there are no reports of trials on transcatheter marking or a study combing the two procedures. Accordingly, it is necessary to first evaluate the safety and reliability of our preoperative marking method, which was developed according to a previous report [14], in a Phase I trial.

## Materials and methods

Trial design and participants

This exploratory prospective study included patients between August 2017 and March 2020 based on the criteria shown in Table 1. Patients were excluded according to the criteria shown in Table 2.

**Table 1:** Inclusion criteria. CT: computed tomography; MRI: magnetic resonance imaging

Inclusion criteria
Hepatectomy was planned for liver tumors measuring ≤20 mm in diameter that could be confirmed on imaging
Eastern Cooperative Oncology Group performance status was 0-1
Age at the time of consent acquisition was ≥20 years
Imaging tests such as CT and MRI showed obvious neoplastic lesions in the liver, which were difficult to identify with ultrasonography at the time of examination or after chemotherapy
The functions of major organs (bone marrow, liver, kidneys, and lungs) were maintained
Cognitive judgment was sufficient to understand the study and provide written consent for participation in this study
Anticoagulant could be withdrawn on the day of marking

**Table 2:** Exclusion criteria.

Exclusion criteria
Serious ischemic heart disease (New York Heart Association classification [15] ≥III)
Severe cirrhosis (liver damage C [16])
Dyspnea requiring oxygen administration due to interstitial pneumonia or pulmonary fibrosis
Chronic renal failure requiring dialysis
Active duplicate cancers that might affect outcomes
Psychosis or psychiatric symptoms
Refusal to participate in the study
Study investigators deeming the patient inappropriate for inclusion

Patient data were collected from one month before marking to one month after surgery. During data collection, we de-identified the data and accessed only the anonymized information during analysis to protect patient privacy.

Sample size estimation

Discussions with the protocol committee at the facility led to the decision that because this was a new protocol that was scientifically valid in other surgical areas but lacked evidence in liver resection and an exploratory study with no proof of safety, including a large number of patients was not desirable; as such, the next phase of the study would be planned after the safety of the protocol was confirmed in about 20 patients.

Treatment protocol

**Figure 1:**
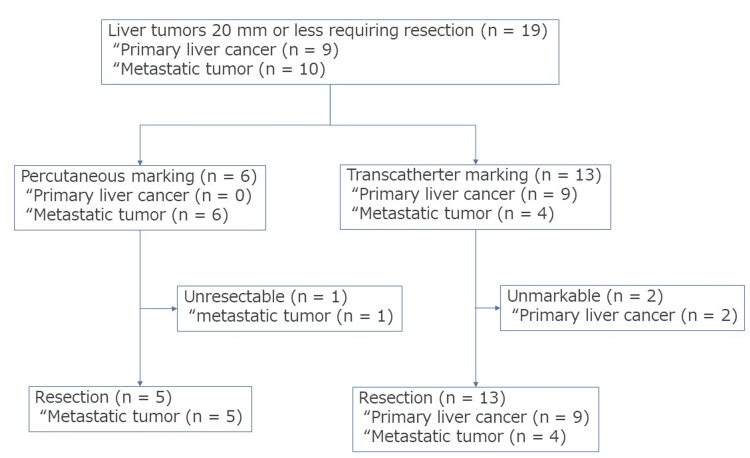
Study flowchart. Hepatectomy was performed in patients with liver tumors measuring 20 mm or less. There were nine cases of primary liver tumor and 10 cases of metastatic tumor. Percutaneous marking was performed in six cases, whereas transcatheter marking was performed in 13 cases. One patient could not undergo resection by percutaneous marking. Conversely, two patients could not undergo transcatheter marking. However, all patients underwent resection.

Marking was performed in the vicinity of the tumor before hepatectomy (Figure 1) using the percutaneous or transcatheter method. Given that the benefit of this procedure to the patient is yet to be proven, marking was performed simultaneously with the examinations for liver tumors. Therefore, additional invasive procedures were avoided by selecting the percutaneous method for patients in whom CT and ultrasound-guided biopsies were performed. The transcatheter method was also performed simultaneously with angiography. If the tumor could be confirmed by plain CT or ultrasound and punctured from the body surface, the percutaneous method was selected; if it could not be confirmed by such methods or was difficult to puncture, the transcatheter method was selected. We avoided predetermining the method and attempted to select the most appropriate method for each case. The marking procedure was performed by an interventional radiology specialist certified by the Japanese Society of Interventional Radiology using either method. The distance between the tumor and the marker was measured using horizontal or coronal CT sections. The presence of marker residue was determined using postoperative CT.

Preoperative chemotherapy

In patients with liver metastasis, chemotherapy was administered after marking [17]. Preoperative chemotherapy was administered according to Japanese guidelines. The regimen consisted of eight courses of the CapeOX + Bmab regimen (capecitabine + oxaliplatin + bevacizumab) for colorectal cancer and four courses of the SOX regimen (tegafur-gimeracil-oteracil potassium + oxaliplatin) for gastric cancer. Chemotherapy response was assessed by CT and/or MRI every four courses according to the Response Evaluation Criteria in Solid Tumors [18].

Percutaneous marking

**Figure 2:**
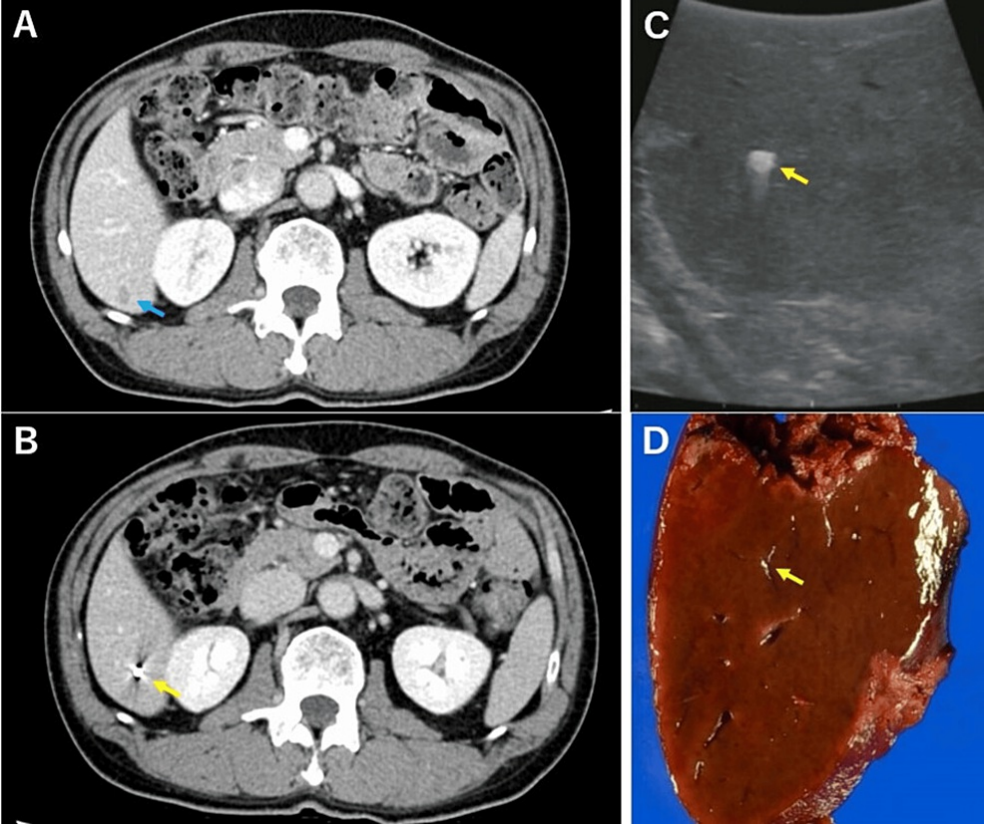
Images from a patient with percutaneous marking. (A) CT before chemotherapy shows a liver tumor (blue arrow). (B) CT before chemotherapy shows the marking coil (yellow arrow). (C) The marking coil can be confirmed by intraoperative echo, and no tumor can be confirmed (yellow arrow). (D) The marking coil can be confirmed in the excised specimen (yellow arrow). CT: computed tomography

Percutaneous marking was performed using local anesthesia under CT/ultrasound guidance. The same site was punctured again following a tumor biopsy for marking. The puncture was performed using a Chiba needle (Cook Medical, Japan). The needle tip was inserted near the tumor (within the range that could be resected at the same time by partial resection), and one microcoil was placed via the needle. The needle was not advanced into the tumor to avoid the risk of dissemination due to direct puncture. The puncture needle was then removed after confirming the absence of internal bleeding. The position of the marker was confirmed by CT before and after its placement (Figure 2). Cases in which tumors can be confirmed by preoperative ultrasound should also be confirmed by IOUS, and thus, they should not be marked; however, clinically, they were selected for tumors that were expected to be eliminated with preoperative chemotherapy.

Transcatheter marking

**Figure 3:**
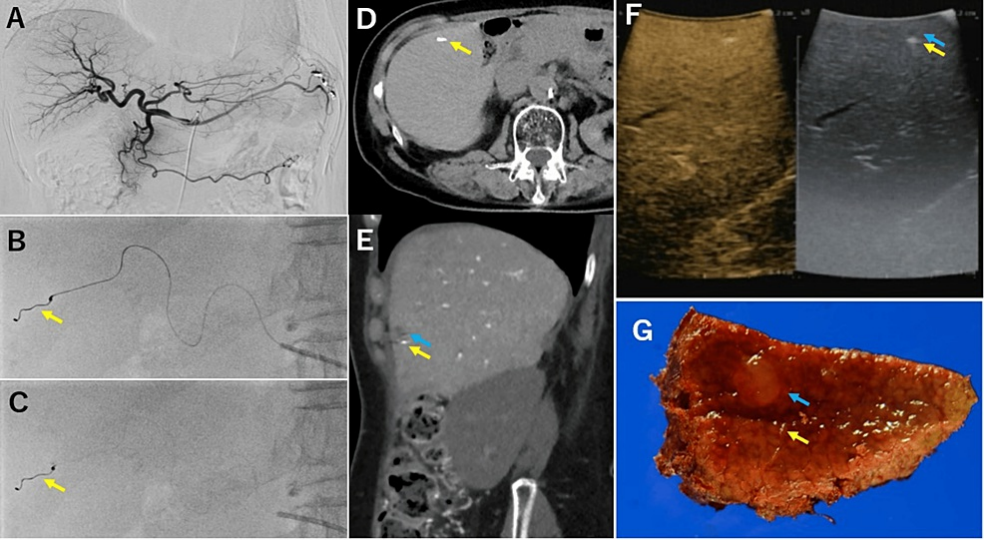
Images from a patient with transcatheter marking. (A-C) At the time of angiography, the hepatic artery near the tumor is marked with a coil (yellow arrow). (D, E) The liver tumor (blue arrow) and marking coil (yellow arrow) can be confirmed on CT. (F) The liver tumor (blue arrow) and marking coil (yellow arrow) can be detected by intraoperative echo. (G) The liver tumor (blue arrow) and marking coil (yellow arrow) are also confirmed in the resected specimens CT: computed tomography

Transcatheter marking was performed following the primary angiography. No new blood vessel puncture was performed for the marking. A 4 Fr catheter was introduced through the femoral artery using the Seldinger technique, and abdominal vessel angiography was performed to identify the feeding artery of the tumor. A microcatheter was inserted superselectively into the vicinity of the tumor (within the range that could be resected at the same time by partial resection). After the positional relationship between the tumor and microcatheter was confirmed via CT, a microcoil was placed. The marker position was then confirmed again via CT (Figure 3).

Liver resection

An IOUS was used to confirm the marker placed preoperatively, and the tumor was resected based on the marker. Per oncological principles, the route of the percutaneous puncture was included in the excision range. Hepatectomy procedures were performed as previously described by Itamoto et al. [19] and Kuroda et al. [20]. In addition, the Pringle maneuver was used during hepatectomy, as necessary. After hepatectomy, the required number of drains was placed where they were required. The drains were removed within one week; however, the duration of drainage was extended if a bile fistula or intra-abdominal abscess occurred. Tumors and markers were searched in all cases using Sonazoid® (common name: perfluorobutane; GE Healthcare Pharma, Tokyo, Japan) contrast ultrasonography.

Outcome measures

The primary outcome measure was the safety of the tumor marking protocol. This was assessed according to the incidence of adverse events and major postoperative complications. The secondary outcome measures were the success rate of tumor resection and changes in blood biochemical parameters.

Ethics approval and consent to participate

The protocol for this research project was approved by a suitably constituted ethics committee at Hiroshima University Hospital (approval number: C-198) and conformed to the provisions of the Declaration of Helsinki. Informed consent was obtained from all study participants.

## Results

A total of 19 patients with liver tumors measuring ≤20 mm and requiring resection were treated between August 2017 and March 2020 (Figure 1). Of these patients, nine (47.3%) had primary liver cancer, whereas 10 (52.7%) had metastatic tumors. Percutaneous marking was performed in six patients, all of whom had metastatic tumors. Transcatheter marking was performed in 13 patients. One patient in the percutaneous group had an unresectable tumor due to tumor growth during post-marking chemotherapy; thus, five patients underwent post-marking resection. In the transcatheter group, marking could not be completed in two patients because of the long distance from the hepatic hilum to the tumor and because of tortuous blood vessels; therefore, the catheter could not be advanced to the tumor. However, all patients underwent resection.

The characteristics of patients in the percutaneous and transcatheter groups are summarized in Table 3 and Table 4, respectively. Additionally, the details of the surgery are presented in Table 5. While the marker should be removed maximally, we intentionally left the remaining marker, as we decided not to remove excess liver tissue (larger than that needed for hepatectomy) to remove the marker because of the distance between the marker and the tumor. The rates of postoperative complications and postoperative liver failure were comparable to those of normal hepatectomy.

**Table 3:** Patient characteristics in the percutaneous group (n = 6). Data are presented as the number of patients or median (range). B/C/NBNC: hepatitis B/hepatitis C/non-B non-C liver disease; CR/PR/SD/PD: complete response/partial response/stable disease/progressive disease; PT: prothrombin time; T-Bil: total bilirubin; ICG-R15: indocyanine green retention rate at 15 minutes

Characteristic	Value
Disease (primary/metastasis)	0/6
Etiology (B/C/NBNC)	0/0/6
Liver cirrhosis (+/-)	0/6
Age (years)	62 (53–70)
Sex (male/female)	3/3
Anticoagulant therapy (yes/no)	1/5
Marking (completion/discontinuation)	6/0
Marking complication (yes/no)	0/6
Preoperative chemotherapy (yes/no)	6/0
Chemotherapy response (CR/PR/SD/PD)	1/3/1/1
Operation (completion/discontinuation)	5/1
Platelet (×10^3^/μL)	219 (158–312)
PT activity (%)	103 (90–137)
T-Bil (mg/dL)	0.65 (0.4–0.7)
Albumin (g/dL)	3.75 (2.9–4.5)
Creatinine (mg/dL)	0.60 (0.56–0.84)
ICG-R15 (%)	7.6 (4.6–25.5)
Child-Pugh classification (A/B)	6/0
Number of tumors	3 (1–14)
Tumor size at diagnosis (mm)	9.5 (8–13)
Tumor size at surgery (mm)	9.5 (0–13)
Distance between the tumor and marker (mm)	10.5 (1–15)

**Table 4:** Patient characteristics in the transcatheter group (n = 13). Data are presented as the number of patients or median (range). B/C/NBNC: hepatitis B/hepatitis C/non-B non-C liver disease; CR/PR/SD/PD: complete response/partial response/stable disease/progressive disease; PT: prothrombin time; T-Bil: total bilirubin; ICG-R15: indocyanine green retention rate at 15 minutes

Characteristic	Value
Disease (primary/metastasis)	9/4
Etiology (B/C/NBNC)	2/5/6
Liver cirrhosis (presence/absence)	3/10
Age (years)	71 (45–83)
Sex (male/female)	12/1
Anticoagulant therapy (yes/no)	3/10
Marking (completion/discontinuation)	11/2
Marking complication (present/absent)	0/13
Preoperative chemotherapy (yes/no)	4/9
Chemotherapy response (CR/PR/SD/PD)	0/3/1/0
Operation (completion/discontinuation)	13/0
Platelet (×10^3^/μL)	160 (53–373)
PT activity (%)	92 (72–116)
T-Bil (mg/dL)	0.6 (0.4–2.3)
Albumin (g/dL)	3.9 (2.6–4.6)
Creatinine (mg/dL)	0.86 (0.55–1.2)
ICG-R15 (%)	13.7 (1.8–30.6)
Child-Pugh classification (A/B)	13/0
Number of tumors	2 (1–7)
Tumor size at diagnosis (mm)	8 (4–22)
Tumor size at surgery (mm)	8 (4–22)
Distance between the tumor and marker (mm)	12 (8–20)

**Table 5:** Surgical and short-term outcomes. Data are presented as the number of patients or median (range). PHLF: post-hepatectomy liver failure

	Percutaneous group	Transcatheter group
Operative method (open/laparoscopy)	4/1	8/5
Operation time (minutes)	363 (136–481)	291 (160–671)
Blood loss (g)	121 (10–722)	486 (15–2,714)
Intraoperative tumor identification (possible/impossible)	4/1	12/1
Intraoperative marker identification (possible/impossible)	5/0	11/0
Tumor resection (completion/discontinuation)	5/0	13/0
Surgical margin (mm)	7.5 (4–15)	7 (0–15)
Residual marker (whole/part/none)	2/0/4	1/3/9
Postoperative complication (yes/no)	0/6	1/12
PHLF (yes/no)	0/6	3/10

## Discussion

Small liver nodules are difficult to distinguish intraoperatively. Tumors that cannot be identified by ultrasonography are challenging to treat locally, with procedures such as radiofrequency ablation; and when resection is performed, it can only be conducted in approximation based on anatomic landmarks. This carries the risk of an unnecessarily large hepatic volume resection or leaving parts of the tumor undetected. Furthermore, although a report of successful percutaneous preoperative marking has been published [10], most tumors cannot be marked percutaneously in clinical practice. It is hypothesized that an adaptable choice of percutaneous marking or transcatheter marking would prove to be useful. To our knowledge, the current study is the first to perform preoperative marking of primary HCC using an intravascular catheter. As a preliminary step to a Phase II trial to demonstrate the usefulness of this method, we were able to establish the safety of this method in a Phase I trial.

Marking metastatic liver cancer using the percutaneous method has been reported previously [21]. In the present study, preoperative tumor marking was successfully performed via the percutaneous and transcatheter approaches. Percutaneous marking was possible in all six patients with metastatic liver tumors. Meanwhile, in the transcatheter group, marking failure only occurred in 2/13 patients. Four patients in this group had metastatic liver tumors and nine had HCC. Both cases with marking failure were of HCC. Small HCCs may be difficult to identify without angiography [22]. In addition, factors such as tumor location (e.g., under the dome, deep in the dome, or near the main vessel) make marking more challenging in the percutaneous method, which could explain the higher rate of marking failure in the transcatheter group, as marking of some HCC tumors could not be performed using the percutaneous method. This suggests that in marking liver tumors, the modalities and approaches that can be used to mark the tumor differ depending on the location and size of the tumor. Therefore, our new method, which allows flexibility in choosing between percutaneous and transcatheter approaches is practical.

Various methods such as indocyanine green (ICG) fluorescence [23] and intraoperative contrast-enhanced ultrasound [24] have been developed for navigation during hepatectomy. However, ICG fluorescence can only be performed on the surface of the liver. Moreover, detecting a small tumor in liver cirrhosis is often difficult using intraoperative contrast-enhanced ultrasound. Marking navigation is a useful technique addressing the limitations of these methods, allowing surgical navigation even beyond the liver surface and in liver cirrhosis.

Nonetheless, the accuracy of marking and the safety of the marker being placed near the tumor are yet to be established. In this study, the percutaneous markers could be placed within 10.5 mm from the tumor, and transcatheter markers could be placed within 12 mm. The farthest distance was 20 mm. This distance between the tumor and the marker is acceptable for effective navigation in actual surgery. Markers were identified by IOUS in all patients, indicating their effectiveness in identifying the excision site. Marking was also highly effective for one patient in whom the tumor disappeared unexpectedly following chemotherapy. The tumor could be resected in all patients with the marker as a guide.

For two patients in the transcatheter group, the catheter could not reach the tumor site because of the distance from the hepatic hilum to the tumor and because of tortuous blood vessels; thus, the catheter could not be advanced to the tumor. One of the two patients had an anatomic variation in which the right hepatic artery diverged from the superior mesenteric artery, which may have contributed to the difficulty in marking. Future studies should investigate alternative methods, such as switching to the percutaneous method, for marking in such cases.

With respect to safety, no marking-related complications occurred in either the percutaneous or transcatheter groups. One patient developed a postoperative complication, but it was unrelated to the preoperative marking. Meanwhile, six marker remnants were found. These showed two patterns. The first pattern was where chemotherapy was not successful after marking and hepatectomy was not indicated due to tumor growth. This pattern was observed in one patient who underwent percutaneous marking. In the second pattern, the markers were not within the resection range because they were far from the tumor. This was found in one patient in the percutaneous group and four patients in the transcatheter group. In the transcatheter method, the coil is left on the central side of the transected part of the vessel because of the straightening of the coil. To avoid such a problem, a shorter coil could be considered in the future. However, the coil used in this method is the one used for embolizing blood vessels and was originally designed on the assumption that it will remain in the body permanently. As such, the remnants may have no adverse impact on organ function. No adverse events related to the residual coil were observed in this case. In addition, even if the marker remains, as it is not too large, only slight artifacts are visible on postoperative CT, and this did not interfere with the diagnosis.

This study has some limitations. As this was an exploratory study and the safety of the protocol could not be confirmed, the number of cases was purposely kept small. Given that safety was not confirmed, marking was performed concurrently with other preoperative examinations such as angiography and liver biopsy. The optimal patients for marking are yet to be determined; hence, patient enrollment based on optimum benefit could not be performed. Although preoperative marking has originally been considered most effective against tumors that are difficult to detect, the evaluation of its efficacy is limited because the study was conducted on detectable tumors. Large-scale Phase II trials are required to further verify the safety and effectiveness of preoperative marking in liver tumors. In fact, given the results of this study, which have provided evidence of the safety of this method, we have currently initiated a Phase II trial to demonstrate its efficacy.

## Conclusions

This study demonstrated the safety and feasibility of preoperative marking for small liver tumors. Preoperative marking for the intraoperative identification of liver tumors does not have any adverse effects and enables surgical navigation, making it a safe and effective modality. These findings provide a basis for improving the complete resection rates in these tumors. To show the efficacy of preoperative marking, a future prospective study will focus on liver tumor cases that are difficult to confirm preoperatively.
